# Mitochondrial Traits Previously Associated With Species Maximum Lifespan Do Not Correlate With Longevity Across Populations of the Bivalve *Arctica islandica*

**DOI:** 10.3389/fphys.2019.00946

**Published:** 2019-07-26

**Authors:** Enrique Rodríguez, Cyril Dégletagne, Tory M. Hagen, Doris Abele, Pierre U. Blier

**Affiliations:** ^1^Département de Biologie, Université du Québec, Rimouski, QC, Canada; ^2^Department of Functional Ecology, Alfred Wegener Institute, Helmholtz Centre for Polar and Marine Research, Bremerhaven, Germany; ^3^CNRS, ENTPE, UMR5023 Laboratoire d’Ecologie des Hydrosystèmes Naturels et Anthropisés, Université Claude Bernard Lyon 1, Villeurbanne, France; ^4^Linus Pauling Institute, Oregon State University, Corvallis, OR, United States

**Keywords:** *Arctica islandica*, bivalve aging model, electron transport system, mitochondria, peroxidation index, reactive oxygen species

## Abstract

The mitochondrial oxidative stress theory of aging posits that membrane susceptibility to peroxidation and the organization of the electron transport system (ETS) linked with reactive oxygen species (ROS) generation are two main drivers of lifespan. While a clear correlation has been established from species comparative studies, the significance of these characteristics as potential modulators of lifespan divergences among populations of individual species is still to be tested. The bivalve *Arctica islandica*, the longest-lived non-colonial animal with a record lifespan of 507 years, possesses a lower mitochondrial peroxidation index (PI) and reduced H_2_O_2_ efflux linked to complexes I and III activities than related species. Taking advantage of the wide variation in maximum reported longevities (MRL) among 6 European populations (36–507 years), we examined whether these two mitochondrial properties could explain differences in longevity. We report no relationship between membrane PI and MRL in populations of *A. islandica*, as well as a lack of intraspecific relationship between ETS complex activities and MRL. Individuals from brackish sites characterized by wide temperature and salinity windows had, however, markedly lower ETS enzyme activities relative to citrate synthase activity. Our results highlight environment-dependent remodeling of mitochondrial phenotypes.

## Introduction

Understanding the physiological determinants of lifespan in animals has been the focus of biologists and gerontologists over the past decades. Comparative studies suggest a role for mitochondrial structure and function associated with the aging process, in particular the management of reactive oxygen species (ROS). This evidence has been integrated into one unifying hypothesis, the “mitochondrial oxidative stress theory of aging” (MOSTA, reviewed in Blier et al., 2017). It points out, among others, the importance of cell membrane composition and particularly the susceptibility of mitochondrial membrane lipids to oxidation peroxidation index (PI) as potentially causative parameters of cellular aging. In addition, modifications of the redox state and stoichiometry of mitochondrial electron transporters appear to play a crucial role for mitochondrial coupling and the rates of ROS formation (Barja, 2007), and hence are important components of the MOSTA.

Membrane lipids are particularly prone to oxidative damage (Gamliel et al., 2008), and the sensitivity of fatty acids (FA) to oxidation by ROS depends on their chemical conformation, especially with respect to the presence of conjugated double bonds. FA chains with no or only one double bond [saturated fatty acids (SFA) and monounsaturated fatty acids (MUFA)] are more resistant to peroxidation than polyunsaturated FA (PUFA), the sensitivity of which increases exponentially with the number of conjugated double bonds (Holman, 1954). The products of FA oxidation (reactive aldehydes) induce autocatalytic progression of lipid peroxidation that can further damage membrane proteins, PUFA chains and DNA. This leads to impaired protein functions and DNA mutations and underlines the crucial role of membrane lipid composition for cellular integrity, with implications for organismal lifespan (Hulbert, 2010).

The strongest association between a physiological trait and divergences in species lifespan has so far been observed for membrane structure. The early evidence of a strong negative correlation between membrane PI and increasing maximum lifespan was reported in vertebrates, specifically in mammals and birds (Hulbert et al., 2007). Later, Munro and Blier (2012) took advantage of the extreme longevity of *Arctica islandica* (the ocean quahog) to corroborate the same association in five species of bivalves (Munro and Blier, 2012). Among these species, the ocean quahog *A. islandica* is the longest-lived non-colonial animal with a record lifespan of 507 years in the North Atlantic (Butler et al., 2013). Munro and Blier (2012) compared gill membrane lipid composition of *A. islandica* with four shorter-lived bivalve species from the shelf areas off the coasts of eastern Canada belonging to the same subclass (heterochonchia), with lifespans ranging from 28 to 106 years. Membrane PI decreased exponentially with increasing longevity, in part due to a lower DHA (docosahexaenoic acid, 22:6, n-3) content in mitochondrial membranes of longer-lived species. Other molecules protecting bivalve cellular membranes against ROS attack include plasmalogens [dimethylacetals (DMA) in their methylated form] and non-methylene interrupted fatty acids (NMI). Plasmalogens act as a ROS scavenger, whereas NMI reduce susceptibility to peroxidation (Engelmann, 2004; Barnathan, 2009) while maintaining fluidity of the membrane. Although no consistent relationship was observed between NMI abundance and MRL, *A. islandica* had higher NMI and plasmalogen levels than the shortest-lived species *Mya arenaria*. The association previously observed in vertebrates therefore, appears to be conserved in a wide range of animal taxa and tighter, at least in bivalves, for mitochondria.

In addition to membrane structure, Munro et al. (2013) found *A. islandica* mantle mitochondrial isolates to exhibit lower rates of net ROS production than two shorter-lived bivalve species. These differences related to the activities of mitochondrial electron transport system (ETS) complexes, including major sites of ROS production (complex I and III) that differed between *A. islandica* and shorter lived species (especially *M. arenaria*), despite similar oxidative phosphorylation (OXPHOS) capacities. Relatively lower activities of CI (NADH oxidoreductase) and CII (succinate dehydrogenase) relative to downstream complex (cytochrome c oxidase) in *A. islandica* suggest a lower degree of reduction at the ETS, resulting in lower overall mitochondrial ROS production without compromising phosphorylation rates (Munro et al., 2013; Blier et al., 2017). This is reminiscent of the lower CI and CII content in calorie-restricted rats and in long-lived drosophila (Ayala et al., 2007; Neretti et al., 2009). Hence, stoichiometric arrangements between upstream and downstream ETS complexes might play an important role in lifespan determination.

From these studies, we can suspect that a low membrane PI and the associated high robustness to ROS attack are prerequisites for reaching long lifespan. On an evolutionary perspective, one obvious question arising from these observations is to which extent membrane variability among populations of one species could set the plasticity and potential evolution of longevity, or associated life history characters. At the intraspecific level, most studies linking lipid composition to metabolism or lifespan have focused on genetically manipulated strains (Brzȩk et al., 2007) with relatively small differences in maximum lifespan, or on laboratory bred animal lines (Valencak and Ruf, 2013). Haddad et al. (2007) found a lower PI in longer-lived honeybee queens than shorter-lived workers, but otherwise no studies have looked at the importance of membrane composition inside a species in a natural setting. The bias of comparing controlled laboratory experiments with physiological processes occurring in nature, could potentially blur some of the real age-related changes (Austad, 2018). In order to test to what extent mitochondrial characters associated to lifespan divergences among species govern the rate of mitochondrial aging within a species, studying different natural populations displaying a wide range of contrasting average lifespans is necessary.

Considerable population-specific variations in MRL are seen in *A. islandica*, both in the western (Kilada et al., 2007) and eastern (Basova et al., 2012) parts of the North Atlantic. One individual of 507 years was found in Icelandic Coast (IC) waters where animals more than 300 years old are regularly encountered (Strahl et al., 2007; Butler et al., 2013). All other populations have much shorter reported lifespan maxima with the shortest MRL of only 40 years reported for the well-studied Baltic Sea population (Basova et al., 2012, and see Table 1). The different *A. islandica* habitats are characterized by wide thermal and salinity windows, but there is no consistent evidence for specific metabolic rate or antioxidant activities in animals from any population of these regions associated to environmental conditions (Basova et al., 2012). On the other hand, environmental factors such as food and sunlight availability (Moss et al., 2017), as well as dredging effort (Thorarinsdóttir et al., 2009) could help explain differences in lifespan. Begum et al. (2018) used one mitochondrial locus (cytochrome b) to prove that, in spite of their vastly differing MRL, all these populations belong to the same species and hence provide an applicable system to test longevity-associated characters at the intraspecific level.

**TABLE 1:** Life history traits of populations of *Arctica islandica* sampled and their maximal reported longevities (MRL), salinity regime characterization according to mean salinity and salinity amplitude, see Basova et al. (2012): marine 33 PSU; marine/coastal 32 PSU/variable; brackish, <30 PSU.

**Population**	**Coordinates**	**MRL (years)**	**Salinity regime**	**References**
Kiel Bay (KB)	54°32.6 N, 10°42.1 E	36	Brackish	Gruber et al., 2015
White Sea (WS)	66°18 N, 33°38 E	53	Brackish	Basova et al., 2012
Kattegat Sea (KA)	56°10 N, 11°48 E	71	Marine/coastal	
German Bight (GB)	54°10 N 7°53 E	150	Marine/coastal	Kerstin Beyer (Alfred Wegener Institute, personal comm.)
Norwegian Coast (NC)	69°39 N, 18°57 E	300	Marine	
Icelandic Coast (IC)	66°02 N, 14°51 W	507	Marine	Butler et al., 2013

The main objective of the present paper is to determine if characters associated to divergences in lifespan among species can dictate the pace of aging among populations of one species. We therefore compared membrane lipid composition, PI, and ETS complex activities across the same *A. islandica* populations from the Atlantic and the brackish coastal seas. We hypothesized that ETS activities and lipid parameters in the populations vary as a function of MRL in the same fashion as found between different bivalve species (Munro and Blier, 2012). We thus predicted to find (1) a decrease in mitochondrial membrane PI with increasing longevity, (2) a higher %PUFA and %DHA in shorter-lived populations, (3) more plasmalogens in longer-lived populations, and (4) a decrease in enzymatic activity for the CI + CIII relative to COX as longevity increases.

## Materials and Methods

Specimens of *A. islandica* from six populations with contrasting MRLs were collected across a geographic gradient of European coastal seas (KB and WS) and from the North Atlantic (see Table 1 for details). Approximately 500 mg of gills and mantle tissues were dissected, and immediately frozen in liquid nitrogen and kept at −80°C before enzymatic and lipid analyses were undertaken. The mantle was chosen as it is the largest somatic tissue and as a means of comparing with a wealth of other studies, while the gills were chosen on the basis of their important respiratory and environmental sensory role. Mitochondrial isolation was carried out on 250 mg of frozen mantle and gills, as previously described (Munro and Blier, 2012). The resulting mitochondrial pellet was resuspended in 150 μl of buffer, and both mitochondria and cellular debris fractions were conserved at −80°C after nitrogen flush for lipid analysis. The abundance of mitochondrial ETSs in both biological fractions was assessed using the NADH-INT (iodonitrotetrazolium) oxidoreduction assay, targeting complexes I and III. The values for the cellular debris pellets were below 1/20th of those in the mitochondrial pellets, and were therefore considered essentially devoid of mitochondria.

Analysis of the phospholipid composition of the mitochondrial and cellular debris fractions was done following the protocol established by Munro and Blier (2012). Separation of phospholipids was done using solid-phase extraction columns (Bond Elut-NH_2_, 500 mg, 3 mL), and *trans*-methylated overnight at 55°C in a 1% H_2_SO_4_ solution in methanol. The resulting fatty acid methyl esters (FAME) and DMA were recovered in 100 μl hexanes and analyzed by GC-FID (Agilent Trace Ultra 100, Thermo Fisher Scientific, Waltham, MA, United States) using a column with a high polarity stationary phase (HP-88, 60 m, 0.25 mm × 0.20 μm, Agilent Technologies Canada, Mississauga, ON, Canada). All solvents used were of ultra-pure grade. Conveniently, calibration of the system was previously done by GC-MS using common FA mix standards (SUPELCO 37 FAME) marine FA standards (SUPELCO) and DMA standards (SIGMA). FA composition-longevity relationships were tested using relative abundance in mol% of individual FA, FA classes (total saturated, monounsaturated, polyunsaturated, DMA and NMI), as well as of different indexes as per Hulbert et al. (2007). The PI was calculated as PI = (0.025 × % monoenoics) + (0.258 × % 20:2 NMI) + (0.32 × % 22:2 NMI) + (1 × % dienoics) + (2 × % trienoics) + (4 × % tetraenoics) + (6 × % pentaenoics) + (8 × % hexaenoics). The unsaturation index (UI) represents the number of double bonds per 100 acyl chains and was calculated as UI = (% monoenoics) + (2^*^% dienoics) + (3^*^% trienoics) + (4^*^% tetraenoics) + (5^*^% pentaenoics) + (6^*^% hexaenoics). Results will be presented as means ± SEM.

Each gill and mantle sample was weighed and homogenized in 6 volumes of cold homogenization buffer (20 mM Tris (hydroxymethyl) aminomethane, 1 mM EDTA, 0.1% Tween 20, pH 7.4) in Precellys Homogenizer 24 (Bertin Technologies) with 2 times 15 s at 5000 rpm at 8°C. Enzymatic activities of CI + III, COX and CS were measured at 8°C using Plate Reader TriStar (Berthold Technologies) and expressed in U.g^–1^ tissue fresh mass. All assays were run in duplicate. All protocols were adapted from Breton et al. (2009). We chose to present CI + III, rather than using the previous annotation “ETS,” since this assay does not measure COX activity, which is a major component of the system.

Differences between groups were assessed using Systat v. 13 software. Variables were first tested for normality using the Shapiro-Wilk test. A between-group principal analysis (PCA) was used to assess the variation in enzymatic activities and membrane lipid composition for individuals belonging to the six populations studied. This analysis consists to running PCA on a dataset, where observations are gathered by user-defined groups to emphasize the between-groups variability in the analysis process (Dolédec and Chessel, 1989). Here groups were built by distinguishing specimens accordingly to their sampling localization. The significance of the proportion of the variability explained by the grouping factor (e.g., population) in between-groups PCA was tested by Monte-Carlo permutation (999 permutations) and were considered significant when the simulated *p* value = 0.001. Between-groups PCA was performed using “ade4” library (Chessel et al., 2004) implemented in R 3.2.1 (R Development Core Team, 2012).

## Results

### Membrane Susceptibility to Peroxidation Index

**FIGURE 1:**
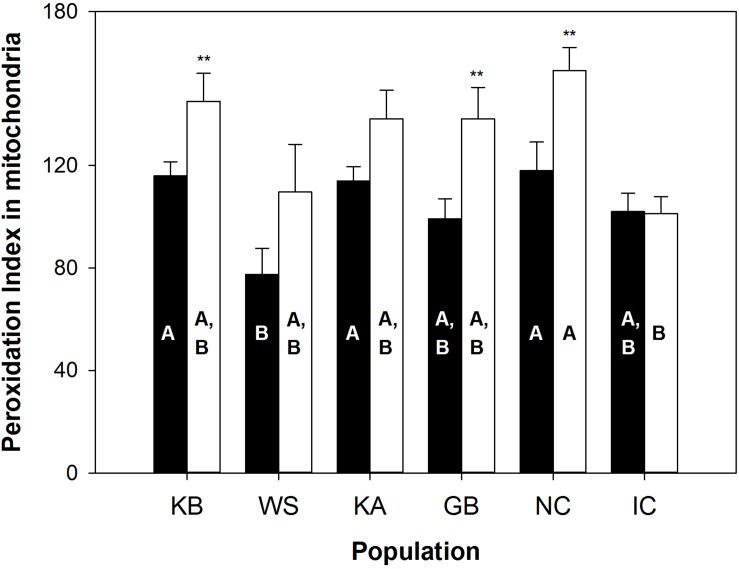
Peroxidation index in mitochondria from *A. islandica* populations ranked from shortest- to longest-lived. Values are means ± SEM. Letters denote significant (*p* ≤ 0.05) differences between populations for gills (filled bars) and mantle (empty bars) tissues, while asterisks indicate significant differences between tissues (^∗∗^*p* ≤ 0.05).

Population grouping significantly explained the variability of lipid peroxidation susceptibility markers (PI, plasmalogens, DHA, n-3 and n-6 PUFA) in the gills (observed variability between populations = 38%, simulated *p* value = 0.001), but not in the mantle tissue (simulated *p* value = 0.085; see Supplementary Figure S1C). The between-population distribution pattern of the lipid markers in the gills, however, did not reflect a clear relationship between PI and population MRL. Instead, WS individuals had a significantly lower PI in the gills than KB (*p* = 0.023), KA (*p* = 0.039), and NC (*p* = 0.019) populations (Figure 1), and did not differ from the IC populations. In all populations except IC, gill mitochondrial membranes had a lower PI than mantle mitochondria. The IC and WS populations had the lowest PIs in both tissues. Gill PI values were 77.5 (± 10.3) and 102.0 (± 7.2) for WS and IC, respectively, while mantle PI values were of 110.0 (± 18.6) and 101.1 (± 6.7) for these same populations (Figure 1). These values related partly to low DHA levels: 4.6% (± 1.0) and 6.2% (± 0.5) in the gills, and 7.5% (± 1.6) and 5.5% (± 0.7) in the mantle, respectively.

The abundance of plasmalogens (measured as their methylation products DMA) and NMIs did not vary significantly among populations. In gill mitochondria, DMA abundance ranged from 12.0% (± 0.9) in WS to 17.7% (± 2.2) in KA, whereas it fluctuated between 8.7% (± 1.7) in WS and 14.0% (± 1.9) in NC in mantle mitochondria. There were significant differences between tissues, as DMA abundance was higher in the gills for KA (*p* = 0.018), GB (*p* = 0.018) and IC (*p* = 0.009) compared to the mantle. Gill mitochondria NMI abundance ranged between 11.6% (± 0.6) in NC to 15.8% in WS, and from 9.1% (± 1.0) in KA to 12.2% (± 2.3) in IC in mantle mitochondria. NMI abundance was also significantly higher in the gills than in the mantle in WS (*p* = 0.036) and KA (*p* = 0.002). See Supplementary Tables S1, S2 for detailed FA composition of mitochondrial membranes in gills and mantle, respectively. FA composition of the cellular debris was similar to that of mitochondria and are thus not presented as figures.

**FIGURE 2:**
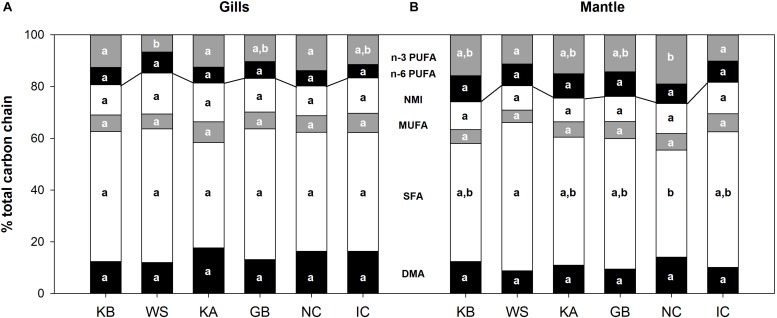
Mitochondrial phospholipid carbon chain composition from *A. islandica* populations ranked from shortest- to longest-lived, in **(A)** gills and **(B)** mantle tissues. Letters denote significant (*p* ≤ 0.05) differences between populations for each carbon chain class: DMA, dimethyl acetals; SFA, saturated fatty acids; MUFA, monounsaturated fatty acids; NMI, non-methylene-interrupted fatty acids; PUFA, polyunsaturated fatty acids. The line delimits peroxidation-resistant (below) and peroxidation-sensitive (above) carbon chains. Samples sizes can be found in Supplementary Table S1.

Between-group PCA did not significantly explain the distribution pattern of phospholipid classes in either gills or mantle. Moreover, the repartition of the amount of phospholipids resistant to peroxidation (NMI, MUFA, SFA, DMA) and those that are peroxidation sensitive (n-6 PUFA and n-3 PUFA) did not differ significantly between populations and could not be linked with their respective MRL (Figure 2).

### ETS Enzyme Activities

The between group PCAs performed on ETS enzyme activities (CI + III, COX; see Supplementary Figures S1A,B) indicates that “population” factor explains significantly more variability in gills (observed variability between population grouping = 61%, simulated *p value* = 0.001; Supplementary Figure S1A) than for mantle tissue (observed variability between population grouping = 26%, simulated *p value* = 0.001; Supplementary Figure S1B). In both cases, the first PCA axis, which accounted for 72 and 84% of the total variance in gills and mantle, respectively, is associated with CI + III and COX activities (normalized to CS activity). The second PCA axis represents 27% of the variability in gills and 15% in mantle tissue based on CI + III⋅COX^–1^ activity.

**FIGURE 3:**
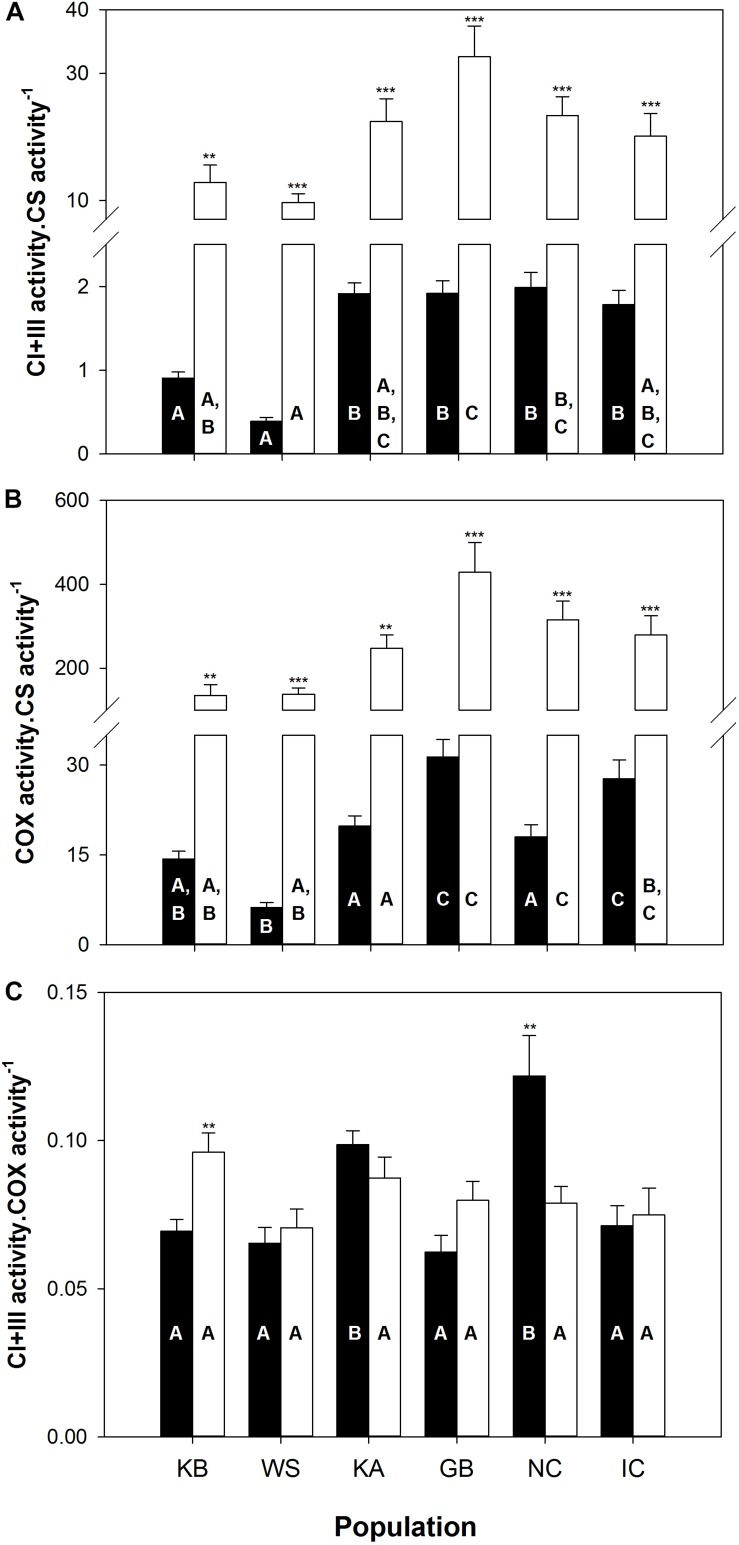
Mitochondrial enzyme activities from sampled *A. islandica* populations ranked from shortest- to longest-lived. **(A)** Complex I and III (CI + III) activity per moles of citrate synthase (CS), **(B)** Cytochrome *c* oxidase (COX) activity normalized by CS, **(C)** CI + III activity normalized by COX. Values are means ± SEM. Letters denote significant (*p* ≤ 0.05) differences between populations for gills (filled bars) and mantle (empty bars) tissues, while asterisks indicate significant differences between tissues (^∗∗^*p* ≤ 0.05, ^∗∗∗^*p* ≤ 0.001).

In both tissues, the brackish water populations from KB and WS exhibit lower CI + III and COX activities, when normalized to CS activity, compared to the fully marine KA, GB, NC and IC. Most comparisons differed significantly (*p* ≤ 0.05; Figures 3A,B), distinguishing populations along the first PCA axis for both tissues. More subtle differences separate the last four populations of which GB and IC animals had higher COX⋅CS^–1^ activity in gills than KA and NC (*p* ≤ 0.05; Figure 3B). Moreover, KA and NC exhibit higher CI + III⋅COX^–1^ ratio in gill mitochondria than all other populations (*p* ≤ 0.05; Figure 3C). These ratio differences can explain the distinction between KA and NC vs. GB and IC along the second axis of the between group PCA in gills. Even though the two shortest-lived populations (KB and WS) exhibited the lowest CI + III⋅CS^–1^ and COX⋅CS^–1^ activities (Figures 3A,B), we could not find any clear correlation between ETS complex stoichiometry and MRL.

Tissue comparisons of CI + III⋅CS^–1^ and COX⋅CS^–1^ revealed significantly lower activities in gills than in mantle mitochondria, for all six populations (*p* ≤ 0.005, Figures 3A,B). As for CI + III⋅CS^–1^, only the KB (*p* = 0.002) and the NC populations (*p* = 0.008) showed significantly higher gill activities compared to the mantle (Figure 3C). Much of these contrasts in metabolic organization observed in short-lived populations, living in brackish water, appear to be driven by significantly higher CS activity (expressed by mg of tissues or proteins) in both tissues (see Supplementary Figure S2).

## Discussion

Mitochondrial resistance to peroxidation and ETS activity linked to ROS production are two important tenets of the MOSTA at the interspecific level (Barja, 2013; Blier et al., 2017). Our study aimed at assessing whether these characters were related to longevity at the intraspecific scale, by taking advantage of the extraordinary MRL diversity across well-studied regional populations of the ocean quahog, *A. islandica*.

### Membrane Lipid Composition Does Not Align With Population MRL

Our results show no MRL related pattern of population specific membrane lipid composition. Instead, differences in membrane lipid composition were very subtle, especially in mantle tissue if we compare them to the interspecific differences in Munro and Blier (Munro and Blier, 2012). Likewise, no relationship between plasmalogen or NMI content and MRL emerged from our analysis. We had predicted lower PI in the longer-lived populations of *A. islandica*, whereas in fact, the short-lived, brackish White Sea population had one of the lowest PIs. Furthermore, neither PUFA nor DHA content (by%) were higher in the shorter-lived populations. Instead, both the WS population, with one of the shortest documented lifespans, and IC subarctic populations with very long lifespan had the lowest %DHA and average %PUFA. Both populations are subpolar and, hence, cold adapted and it is interesting that they have on average lower %PUFA in the population comparison instead of relatively higher unsaturation levels to maintain membrane fluidity in their low temperature habitats [see homeoviscous adaptation in poikilotherms, summarized in Storelli et al. (1998)]. Other important parameters that could be implicated in the aging phenotype of these populations, such as phospholipid classes, sphingolipid or cholesterol content were not included in our analysis and should be assessed in subsequent studies.

Inferring from the lipofuscin content for a standardized 50 years old individual across all *A. islandica* populations, Basova et al. (2017) reported higher rates of fluorescent age pigment, lipofuscin accumulation in the mantle and gills of short-lived (WS, KB) individuals compared to the IC population. Since this pigment is a proxy of physiological aging (Lushchak et al., 2011) and the KB population showed the fastest accumulation of the pigment, followed by the WS, Basova et al. (2017) suggested a high rate of aging in these populations induced by the cellular stress response to large seasonal salinity and temperature amplitude in both brackish habitats. Our results also show similarities between these two brackish sites, with only the PI in gill membranes being markedly lower in WS than KB animals (via lower n-3 PUFA abundance), whereas for all the other longevity-linked lipids, the distribution of the two populations were overlapping in the PCA.

### Ratios of ETS and TCA Cycle Components Differ Among Populations From Different Salinity Backgrounds

In line with our results for membrane composition, the capacities of the ETS compounds, and the ratios between different complexes showed no clear relationship with population MRL. Based on the findings from the interspecific comparison (Munro et al., 2013), we expected decreased CI + III⋅CS^–1^ or CI + III⋅CIV^–1^ activities with increasing population MRL. Instead, tissues of short-lived KB and WS quahogs exhibited lower activities for both mitochondrial ETS complexes than longer-lived populations, when normalized to CS activity as a marker for mitochondrial volume density and TCA cycle units. In fact, the large diversity in enzymatic complex activities between populations (up to 3 and 4-fold difference for CI + III⋅CS^–1^ and COX⋅CS^–1^, respectively) resulted mainly from higher CS activity in short-lived KB and WS populations (Supplementary Figure S2). Hence, contrary to the interspecific comparison, the intraspecific comparison does not support the functional concept of lower upstream (CI + III) *vs.* downstream (COX) ETS activity, but reveals an altered balance between ETS and CS complexes in shorter lived *A. islandica* populations. It should, however, be noted that we did not assess the activity of complex II, another electron entry portal (succinate) and potential source of ROS (Quinlan et al., 2012), which was shown to have a much lower activity in longer-lived bivalves (Munro et al., 2013).

Relatively higher mitochondrial volume density and more TCA cycle units (such as CS) in tissues of short-lived, brackish water *A. islandica* could result in a higher capacity to generate NADH in the TCA cycle in relation to the capacity to pass electrons down the respiratory system toward COX reduction. Zurburg and De Zwaan (1981) highlighted the fact that (hyper-) osmotic stress causes a general shift toward the usage of anaerobic pathways in marine bivalves. Higher aerobic capacity in brackish water could, however, help adjust the free amino acid pool by the TCA cycle (glutamate oxidation) and deliver NADH and aspartate for the aspartate–alanine pathway in the cytosol. Frequent alterations between aerobic and anaerobic glycolysis in bivalves exposed to the vagaries of environmental salinity fluctuations typical for Kiel Bight and the White Sea presumably cause higher metabolic demand, oxidative stress and mitochondrial turnover and may be causal for the faster rates of lipofuscin accrual from deteriorating mitochondria in these populations (Rivera-Ingraham and Lignot, 2017). It appears therefore likely that these biochemical peculiarities are determinant in the “fast aging phenotype” in both brackish *A. islandica* populations.

Thus, mitochondrial energetics may play a role in shortening lifespan of the ocean quahog in brackish water environments, and our study suggest a potential role of mitochondria in the aging process of populations, related to their role in osmoregulation. It remains to say that mitochondrial ETS parameters and membrane PI, in all populations of *A islandica* that we analyzed, are very low compared to other bivalves (Munro and Blier, 2012). In the same line of evidence, Strahl et al. (2007) showed that Icelandic *A. islandica* display one of the lowest growth constants in a comparison of 147 worldwide bivalve populations. This suggests that *A. islandica* has evolved as a long-lived phenotype (see also Moss et al., 2016), including a peroxidation-protected membrane lipid composition across all populations. Recent data (Begum et al., 2018) show very low genetic differences between *A. islandica* from the same sites as in our study, suggesting an important degree of phenotypic plasticity instead of local genetic adaptation. Population-specific maximum age seems therefore to be a function environmental challenge, biotic and/or abiotic, including salinity stress in brackish populations. Similarly, Basova et al. (2012) failed to link population-specific antioxidant enzyme capacities and metabolic rate to MRL (MLSP in Basova et al., 2012), and instead found lower antioxidant activity (superoxide dismutase) in habitats with highly variable annual salinity amplitudes. Additionally, higher DNA damage accumulation rates have been reported in the KB compared to the IC population by Gruber et al. (2015). All these aspects underline the strong influence of adaptation to vastly differing habitat conditions across different populations of this species.

### Tissue Specific Differences and Dietary Influence on Mitochondrial Membranes and ETS Activities

We found important differences between the two tissues, mantle and gills. The lower PI in the gills compared to the mantle tissue in all populations, except the extremely long-lived IC, suggests that the respiratory organs, directly exposed to bottom water oxygen levels during ventilation, might be particularly protected from peroxidation. In addition, gills are exposed to fluctuations of salinity, environmental toxins and pathogens, and appear more exposed and susceptible in a general sense. Consequently, the intensity of apoptotic cell removal in the gills is higher than in mantle tissue of *A. islandica*, as found in the GB and IC populations (Strahl and Abele, 2010). Gill tissue was found to have a higher mitotic index than the mantle in the oyster (Jemaà et al., 2014), and this could translate into differences in accumulation rates of dysfunctional lipids, proteins and DNA. Hence, tissue-specific functions and turnover rates are important to consider when looking at the biochemical determinants of lifespan.

Lifespan in bivalves is strongly linked to latitude (Moss et al., 2016), with longer-lived species and populations generally found at higher latitudes. This could be explained through the physiological effects of lower temperature and light, and hence limited and seasonal food supply at high latitudes, causing prolonged periods of caloric restriction (Moss et al., 2017). As the effect of FA regime on mitochondrial membrane lipids are well established (Guderley et al., 2008; Lemieux et al., 2008), diet might also explain part of the inter-population differences we observed. Few studies have demonstrated an effect of diet on mitochondrial membrane lipid composition in bivalves. Comparing different algal diets of the Pacific oyster *Crassostrea gigas*, Dudognon et al. (2014) found changes in various FA classes, especially in DHA and EPA (eicosapentaenoic acid, 20:5, n-3) in gill mitochondria, which, however, did not cause alterations in gill COX activity, state 3 and state 4 oxygen consumption, or ROS production between diet groups. When studying the effects of diet abundance, temperature and age on the lipid composition of *A. islandica* and the shorter-lived *Spisula solidissima* mitochondria, Munro and Blier (Munro and Blier, 2015) found that although proportions of PUFA and PI increased in both species through microalgae supplementation, the differences between the two in longevity-related parameters (PI and NMI) remained unchanged. These elements suggest that mechanisms regulating membrane composition should be important (reviewed in Hulbert et al., 2014), and the effects of membrane composition modulation on enzymatic activity are not consistently seen across phyla (Lemieux et al., 2008). In the interpretation of our data, a limitation should be considered due to the fact that mitochondria were isolated from frozen tissue (a more uncommon procedure, but see for example Hulbert et al., 2008). Nonetheless, nutrition and abiotic factors in the field likely impact mitochondrial phenotype and function, and form part of the metabolic response to environmental food levels which may also affect life-history traits in marine invertebrates.

At the interspecies level, these environmental conditions could also impact the pace of aging, but on the long term they may not be as critical in setting maximal lifespan that can be reached by a species. For example, Munro et al. (2013) compared five species living in relatively similar environments and comparable optimal and critical temperatures, but with widely divergent maximum lifespans.

## Conclusion

The species *A. islandica* has evolved a long-life phenotype with adjustment of mitochondrial membrane composition, low metabolic activity and control of cellular waste products (Strahl et al., 2007; Strahl and Abele, 2010; Munro et al., 2013; Moss et al., 2016). Our results show that at the intraspecific level, there is no direct relationship between two important mitochondrial components: FA composition of the membrane, enzymatic organization at the level of CI + III, COX and CS, and population-specific MRL. Nonetheless, it appears that contrary to the ratio of upstream to downstream ETS complexes (which is conserved among populations), the enzymatic activities and aerobic capacities (as citrate synthase activity per mg of proteins) are plastic traits among populations. Brackish, coastal environments in KB and WS is associated to an increase of citrate synthase maximal activity relative to ETS capacities suggesting elevated rates of substrate oxidation. This might better support both osmoregulation and anaerobic energy metabolism under fluctuant salinity and temperature conditions in these habitats.

Differences in population longevity appear to be independent of sampling effort (see for example Gruber et al., 2015) and, although no precise record of age at maturity from our sites could be found, data from the western Atlantic suggests that it is variable among populations (Thompson et al., 1980) and likely correlated to longevity as it is in different bivalve species (Ridgway et al., 2011). As our understanding of oxidative stress increases, it appears that mitochondrial ROS management is at the center of modulation of life-history traits (such as reproduction and growth, see Costantini, 2018). We then suggest that the divergences in observed MRL among populations should not only be the consequence of detrimental effects of stressful conditions, but could also result from the adjustment of maturation to insure completion of the life cycle in the different environmental conditions experienced by the populations. It seems that *A. islandica* populations are able to deal with a large range of environmental conditions at the expense of MRL. We suspect that all *A. islandica*, independently of the population they belong to, appear to have the potentiality to reach extreme longevity. However, we cannot exclude that reaching such high longevity as 507 years old would require very special dispositions, even for a centenarian bivalve, as particular environmental or ecological conditions (such as predation pressure, see Moss et al., 2017), specific mitochondrial phenotypes and/or peculiar genetic predispositions.

The results of the present study clearly reveal that a characters that have been tightly associated to divergences of lifespan among species namely PI of mitochondrial membranes cannot explain the observed divergences among populations of the longest-lived species *A. islandica*. It therefore rules out the proposal that variation in PI could alone manage pace of aging or dictate the expressed lifespan of populations. High metabolic demands requested by stressful and viable environments could partly explain these populations’ longevity divergences, but they cannot only result from the impact of stress on physiological conditions, since divergences in lifespan occur with adjustments of the age at reproduction. These bivalves are thus a useful system to disentangle the metabolic characters modulating reproduction and linking maturation to lifespan and provide support or challenge aging theories. Integrating field observation and experimentation with results obtained in the laboratory is the only way to tackle this crucial question (Austad, 2018).

Future studies shall investigate the biochemical adjustments in mitochondria under range edge conditions, including their response to fluctuating salinities and hypoxia in terms of ROS production and the use of alternative electron transport pathways. Other mitochondrial determinants of these lifespan differences have been proposed in recent studies and should be explored. These potential candidates include different phospholipid species such as cardiolipin (reviewed in Paradies et al., 2010), the stability of ETS supercomplexes (Gómez and Hagen, 2012), the control of electron flux and ROS management (Blier et al., 2017; Munro and Treberg, 2017), and the rates of mtDNA mutations (reviewed in Pinto and Moraes, 2015).

## Data Availability

All datasets generated for this study are included in the manuscript and/or the Supplementary Files.

## Author Contributions

ER and CD performed the experiments, analyzed the data, and drafted the manuscript. PB, DA, and TH aided in interpreting the results and writing the manuscript. ER and PB planned the experimentation. ER performed lipid biochemistry, statistical, and enzymatic analyses, wrote the first version of the manuscript, and participated in the corrections. CD participated in the sampling, and performed some of the enzymatic analysis. DA provided the samples. CD, TH, DA, and PB participated in the redaction and correction of the manuscript.

## Conflict of Interest Statement

The authors declare that the research was conducted in the absence of any commercial or financial relationships that could be construed as a potential conflict of interest.
