# Supplementary material for: Mitochondrial Traits Previously Associated With Species Maximum Lifespan Do Not Correlate With Longevity Across Populations of the Bivalve Arctica islandica
Source: Front Physiol. 2019 Jul 26;10:946. doi: 10.3389/fphys.2019.00946 (PMC6676799; doi:10.3389/fphys.2019.00946)
Supplement: Supplementary file 1 [file Data_Sheet_1.docx]

***Supplementary material***


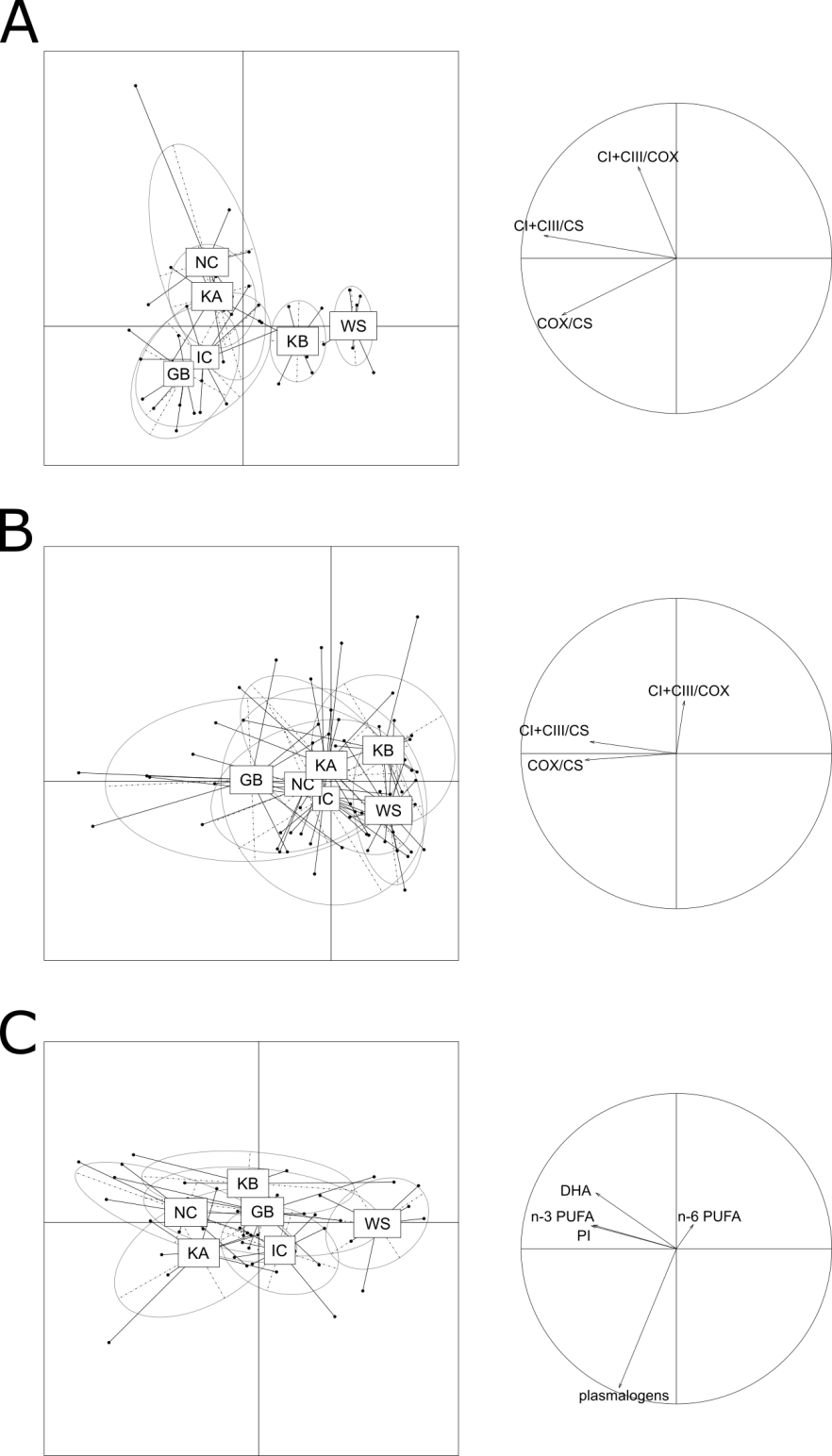


**Supporting figure S1.** Between group principal component analyses (PCA) on European populations (KB: Kiel Bay, KA: Kattegat, GB: German Bight, WS: White Sea, NC: Norwegian Coast and IC: Iceland) of *Arctica islandica* according to enzymatic activities in the gills (A), enzymatic activities in the mantle (B) and membrane lipids linked to longevity in the gills (C). Circle at the right of the plot describes the physiological meaning of axes 1 and 2 of between-groups PCA.

**Supporting figure S2.** (A) Citrate synthase activity normalized by tissue fresh weight and (B) by mg of proteins from sampled *A. islandica* populations ranked from shortest- to longest-lived. Values are means ±SEM. Letters denote significant (*p* ˂ 0.05) differences between populations for gills (filled bars) and mantle (empty bars) tissues, while asterisks indicate significant differences between tissues (**: *p* ˂ 0.05, ***: *p* ˂ 0.001).


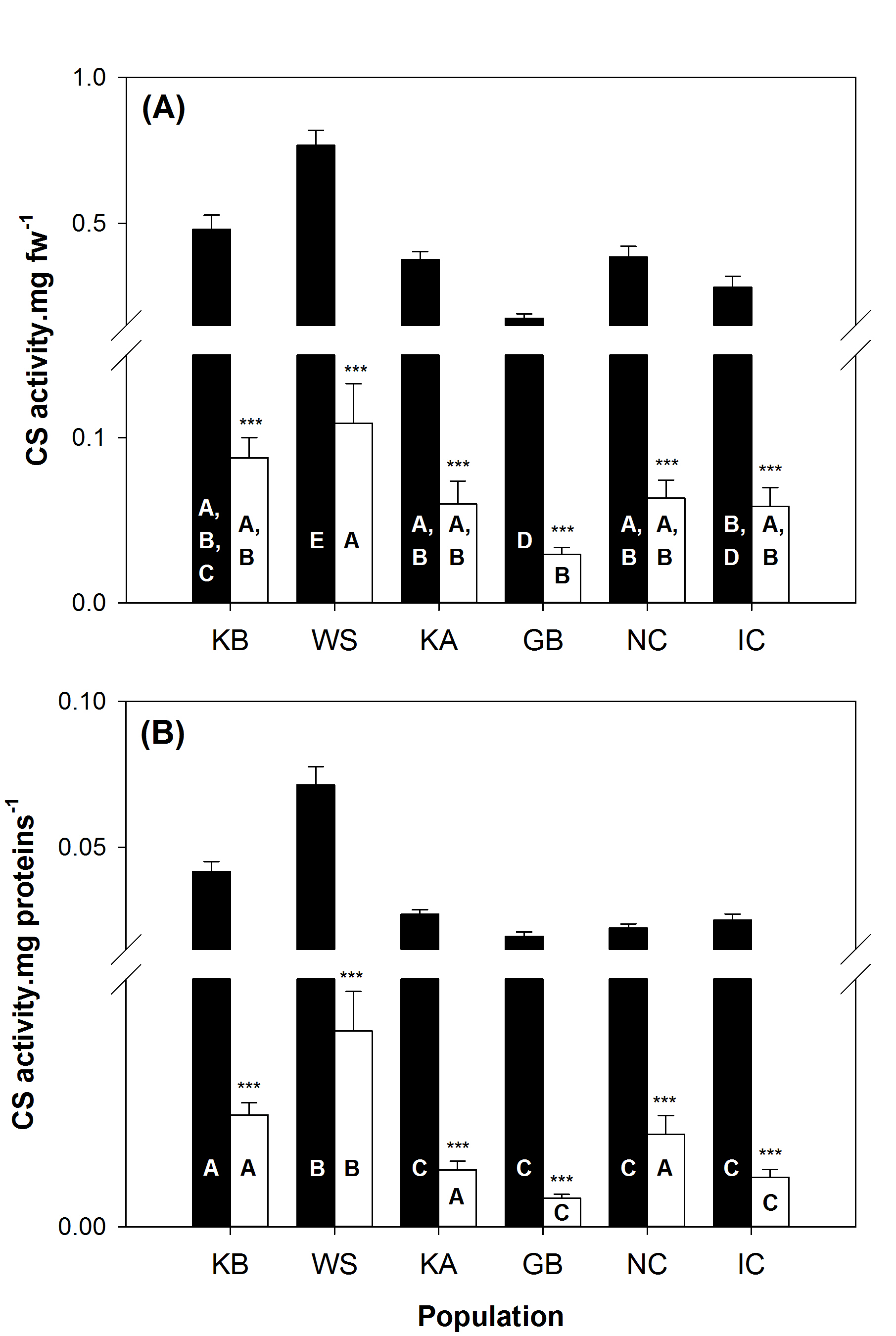


**Table S1**. Fatty acid and DMA composition (mol%) of phospholipids from gill mitochondria in european populations of *Arctica islandica*

|  | Kiel Bay (n = 9) | White Sea (n = 8) | Kattegat Sea (n = 9) | German Bight (n =9) | Norwegian Coast (n = 8) | Icelandic Coast (n = 8) |
| --- | --- | --- | --- | --- | --- | --- |
| 9:0 | 1.0 ± 0.3 | 2.1 ± 1.0 | 1.0 ± 0.3 | 2.0 ± 0.6 | 0.6 ± 0.1 | 1.0 ± 0.3 |
| 14:0 | 1.1 ± 0.2 | 1.5 ± 0.3 | 0.9 ± 0.1 | 1.3 ± 0.4 | 1.0 ± 0.1 | 0.9 ± 0.2 |
| 15:0 | 0.6 ± 0.1 | 0.5 ± 0.1 | 0.4 ± 0.1 | 0.6 ± 0.2 | 0.5 ± 0.1 | 0.4 ± 0.1 |
| 16:0 | 21.4 ± 1.8 | 18.6 ± 1.9 | 17.3 ±2.2 | 20.3 ± 2.4 | 19.4 ± 1.7 | 17.3 ± 1.3 |
| 17:0 | 0.8 ± 0.1 | 0.8 ± 0.1 | 0.9 ± 0.1 | 0.9 ± 0.1 | 1.1 ± 0.0 | 1.3 ± 0.1 |
| 18:0 | 22.3 ± 1.4 | 24.8 ± 2.9 | 17.6 ± 1.8 | 22.3 ± 2.1 | 19.9 ± 1.9 | 22.6 ± 2.2 |
| 18:1 n-13 | 2.1 ± 0.1 | 2.5 ± 0.3 | 3.9 ± 0.7 | 2.7 ± 0.4 | 2.2 ± 0.2 | 3.3 ± 0.3 |
| 18:1 n-9 | 1.6 ± 0.2 | 1.5 ± 0.2 | 1.4 ± 0.2 | 1.7 ± 0.2 | 1.5 ± 0.2 | 1.4 ± 0.1 |
| 18:1 n-7 | 0.6 ± 0.1 | 0.4 ± 0.1 | 0.9 ± 0.1 | 0.4 ± 0.1 | 0.9 ± 0.1 | 1.2 ± 0.2 |
| 18:2 n-6 | 0.5 ± 0.2 | 0.2 ± 0.1 | 0.3 ± 0.1 | 0.2 ± 0.1 | 0.4 ± 0.1 | 0.2 ± 0.1 |
| 20:1 n-11 | 1.1 ± 0.6 | 0.2 ± 0.1 | 0.2 ± 0.0 | 0.6 ± 0.3 | 0.3 ± 0.1 | 0.3 ± 0.1 |
| 20:1 n-9 | 0.2 ± 0.1 | 0.4 ± 0.2 | 0.4 ± 0.1 | 0.2 ± 0.0 | 0.3 ± 0.0 | 0.2 ± 0.1 |
| 20:1 n-7 | 1.0 ± 0.0 | 0.8 ± 0.1 | 1.1 ± 0.2 | 0.9 ± 0.1 | 1.1 ± 0.2 | 1.0 ± 0.1 |
| 20:2 n-6 | 0.7 ± 0.0 | 0.7 ± 0.1 | 0.7 ± 0.2 | 0.5 ± 0.0 | 0.9 ± 0.2 | 0.5 ± 0.1 |
| 20:3 n-6 | 2.0 ± 1.0 | 4.6 ± 1.6 | 2.3 ± 0.3 | 2.5 ± 1.2 | 2.1 ± 0.3 | 2.1 ± 0.5 |
| 20:4 n-6 | 1.7 ± 0.4 | 1.2 ± 0.2 | 1.7 ± 0.3 | 1.6 ± 0.2 | 1.3 ± 0.3 | 1.3 ± 0.2 |
| 20:5 n-3 | 3.6 ± 0.3 | 1.2 ± 0.4 | 3.8 ± 0.3 | 3.0 ± 0.5 | 5.0 ± 1.2 | 4.0 ± 0.3 |
| 22:4 n-6 | 1.1 ± 0.3 | 0.6 ± 0.1 | 0.7 ± 0.1 | 1.0 ± 0.2 | 0.5 ± 0.2 | 0.5 ± 0.1 |
| 22:5 n-6 | 0.7 ± 0.3 | 0.8 ± 0.2 | 0.5 ± 0.1 | 0.7 ± 0.2 | 0.7 ± 0.2 | 0.6 ± 0.1 |
| 22:5 n-3 | 0.4 ± 0.2 | 0.6 ± 0.1 | 1.0 ± 0.2 | 0.8 ± 0.2 | 1.0 ± 0.1 | 0.8 ± 0.2 |
| 22:6 n-3 | 8.1 ± 0.5 | 4.6 ± 1.0 | 7.4 ± 0.5 | 6.0 ± 0.6 | 7.1 ± 0.9 | 6.2 ± 0.5 |
| NMID 20:2 (Δ5, 11) | 7.1 ± 0.5 | 6.3 ± 1.6 | 7.6 ± 1.2 | 7.1 ± 0.7 | 6.7 ± 1.0 | 8.2 ± 0.6 |
| NMID 20:2 (Δ5, 13) | 1.6 ± 0.1 | 2.2 ± 0.3 | 3.5 ± 0.7 | 1.7 ± 0.1 | 2.0 ± 0.5 | 2.1 ± 0.3 |
| NMID 22:2 (Δ7, 13) | 1.1 ± 0.1 | 0.6 ± 0.1 | 0.9 ± 0.1 | 0.5 ± 0.1 | 0.6 ± 0.1 | 0.9 ± 0.1 |
| NMID 22:2 (Δ7, 15) | 0.3 ± 0.1 | 0.4 ± 0.2 | 0.0 ± 0.0 | 0.2 ± 0.1 | 0.2 ± 0.1 | 0.2 ± 0.1 |
| NMIT 22:3 (7, 13, 16) | 1.5 ± 0.8 | 4.5 ± 1.3 | 2.1 ± 0.8 | 2.5 ± 1.1 | 1.7 ± 0.3 | 1.9 ± 0.4 |
| DMA 16:0 | 0.2 ± 0.1 | 0.2 ± 0.1 | 0.1 ± 0.0 | 0.2 ± 0.0 | 0.1 ± 0.0 | 0.2 ± 0.1 |
| DMA 17:0 iso II | 0.0 ± 0.0 | 0.1 ± 0.0 | 0.0 ± 0.0 | 0.0 ± 0.0 | 0.1 ± 0.0 | 0.0 ± 0.0 |
| DMA 17:0 | 0.1 ± 0.0 | 0.1 ± 0.0 | 0.0 ± 0.0 | 0.0 ± 0.0 | 0.1 ± 0.0 | 0.1 ± 0.0 |
| DMA 18:0 iso I | 0.7 ± 0.3 | 0.3 ± 0.2 | 0.6 ± 0.2 | 0.8 ± 0.2 | 0.7 ± 0.3 | 0.6 ± 0.2 |
| DMA 18:0 | 1.0 ± 0.5 | 1.7 ± 0.3 | 3.4 ± 0.5 | 2.2 ± 0.7 | 2.9 ± 0.5 | 2.4 ± 0.6 |
| DMA 19:0 iso | 0.3 ± 0.1 | 0.8 ± 0.2 | 0.5 ± 0.1 | 0.3 ± 0.1 | 0.5 ± 0.1 | 0.5 ± 0.1 |
| DMA 20:1 iso II | 0.0 ± 0.0 | 0.0 ± 0.0 | 0.0 ± 0.0 | 0.0 ± 0.0 | 0.0 ± 0.0 | 0.0 ± 0.0 |
| DMA 20:1 | 9.6 ± 0.5 | 7.6 ± 1.0 | 12.4 ± 1.5 | 9.1 ± 0.8 | 10.2 ± 1.0 | 12.0 ± 0.8 |
| Branched FA | 2.6 ± 0.3 | 1.9 ± 0.4 | 2.6 ± 0.2 | 2.1 ± 0.5 | 3.2 ± 0.5 | 1.7 ± 0.3 |
| Fatty acids ˂ 0.5% | 2.3 ± 0.3 | 4.5 ± 1.4 | 3.1 ± 0.4 | 3.4 ± 0.9 | 3.4 ± 0.4 | 3.5 ±0.3 |
| SFA | 50.2 ± 2.3 | 51.7 ± 3.7 | 40.7 ± 3.9 | 50.5 ± 3.0 | 46.0 ± 2.8 | 46.0 ± 3.0 |
| MUFA | 6.5 ± 0.7 | 5.7 ± 0.7 | 8.0 ± 0.8 | 6.5 ± 0.5 | 6.4 ± 0.4 | 7.4 ± 0.2 |
| PUFA | 31.0 ± 1.7 | 30.7 ± 3.0 | 33.6 ± 2.1 | 29.9 ± 2.0 | 31.3 ± 1.8 | 30.3 ± 1.8 |
| n-6 PUFA | 6.7 ± 1.0 | 8.1 ± 1.5 | 6.1 ± 1.0 | 6.5 ± 1.2 | 5.9 ± 0.6 | 5.1 ± 0.5 |
| n-3 PUFA | 12.6 ± 0.8 | 6.7 ± 1.5 | 12.5 ± 0.8 | 10.4 ± 1.2 | 13.9 ± 1.7 | 11.5 ± 0.9 |
| n-3 PUFA (%PUFA) | 41.2 ± 2.4 | 22.2 ± 3.7 | 37.6 ± 2.4 | 35.3 ± 3.8 | 43.7 ± 3.7 | 38.0 ± 1.7 |
| PUFA (without NMI) | 19.3 ± 1.1 | 14.8 ± 1.9 | 18.6 ± 1.3 | 16.9 ± 1.4 | 19.8 ± 1.7 | 16.7 ± 1.2 |
| NMI total | 11.7 ± 0.8 | 15.8 ± 2.3 | 15.0 ± 1.2 | 13.0 ± 1.1 | 11.6 ± 0.6 | 13.7 ± 0.9 |
| NMI (%PUFA) | 37.6 ± 1.1 | 51.0 ± 4.1 | 44.4 ± 2.0 | 43.8 ± 2.7 | 37.5 ± 2.2 | 45.1 ± 1.6 |
| DMA ˂ 0.5% | 0.1 ± 0.1 | 0.2 ± 0.2 | 0.0 ± 0.0 | 0.2 ± 0.1 | 0.0 ± 0.0 | 16.3 ± 1.4 |
| Branched DMA (iso) | 1.2 ± 0.4 | 1.9 ± 0.2 | 1.5 ± 0.3 | 1.4 ± 0.4 | 1.7 ± 0.6 | 1.2 ± 0.2 |
| DMA total | 12.3 ± 1.0 | 12.0 ± 0.9 | 17.7 ± 2.2 | 13.1 ± 1.3 | 16.3 ± 1.9 | 16.3 ± 1.4 |
| Unsaturation index | 135.2 ± 6.3 | 115.9 ± 10.8 | 143.4 ± 7.6 | 125.4 ± 7.4 | 137.5 ± 9.0 | 130.6 ± 7.7 |
| Peroxidation index | 116.0 ± 5.5 | 77.5 ± 10.3 | 113.9 ± 5.7 | 99.2 ± 7.8 | 117.9 ± 11.2 | 102.0 ± 7.2 |
| MRL (years) | 36 | 53 | 71 | 150 | 300 | 507 |

**Table S2**. Fatty acid and DMA composition (mol%) of phospholipids from mantle mitochondria in european populations of *Arctica islandica*

|  | Kiel Bay (n = 9) | White Sea (n = 7) | Kattegat Sea (n = 9) | German Bight (n = 9) | Norwegian Coast (n = 9) | Icelandic Coast (n = 8) |
| --- | --- | --- | --- | --- | --- | --- |
| 9:0 | 1.2 ± 0.2 | 1.23 ± 0.3 | 1.6 ± 0.7 | 1.2 ± 0.3 | 0.7 ± 0.2 | 1.8 ± 0.8 |
| 14:0 | 1.0 ± 0.2 | 1.7 ± 0.3 | 0.8 ± 0.2 | 1.5 ± 0.2 | 1.0 ± 0.1 | 1.5 ± 0.2 |
| 15:0 | 0.6 ± 0.1 | 0.7 ± 0.2 | 0.5 ± 0.1 | 0.9 ± 0.1 | 0.6 ± 0.1 | 0.7 ± 0.1 |
| 16:0 | 18.7 ± 2.0 | 25.1 ± 3.1 | 18.6 ± 1.2 | 19.9 ± 1.5 | 17.4 ± 1.6 | 20.5 ± 2.0 |
| 17:0 | 1.0 ± 0.1 | 0.8 ± 0.1 | 0.8 ± 0.1 | 0.9 ± 0.1 | 1.1 ± 0.1 | 1.0 ± 0.1 |
| 18:0 | 20.4 ± 2.0 | 25.3 ± 3.1 | 24.6 ± 1.9 | 23.2 ± 2.4 | 18.5 ± 1.2 | 25.4 ± 2.4 |
| 18:1 n-13 | 1.6 ± 0.2 | 0.9 ± 0.2 | 1.5 ± 0.2 | 1.3 ± 0.1 | 1.9 ± 0.2 | 1.5 ± 0.3 |
| 18:1 n-9 | 2.0 ± 0.2 | 1.9 ± 0.2 | 2.0 ± 0.1 | 2.8 ± 0.4 | 2.2 ± 0.2 | 1.8 ± 0.1 |
| 18:1 n-7 | 0.6 ± 0.1 | 0.6 ± 0.2 | 0.8 ± 0.1 | 0.7 ± 0.3 | 1.0 ± 0.1 | 0.8 ± 0.1 |
| 18:2 n-6 | 0.4 ± 0.2 | 0.3 ± 0.1 | 0.5 ± 0.3 | 0.3 ± 0.1 | 0.6 ± 0.2 | 0.5 ± 0.2 |
| 20:1 n-11 | 0.2 ± 0.2 | 0.5 ± 0.4 | 0.4 ± 0.2 | 0.6 ± 0.3 | 0.2 ± 0.1 | 1.0 ± 0.9 |
| 20:1 n-9 | 0.4 ± 0.2 | 0.2 ± 0.0 | 0.4 ± 0.1 | 0.2 ± 0.1 | 0.2 ± 0.1 | 0.2 ± 0.0 |
| 20:1 n-7 | 0.7 ± 0.1 | 0.7 ± 0.1 | 0.9 ± 0.1 | 1.0 ± 0.1 | 0.9 ± 0.1 | 0.9 ± 0.1 |
| 20:2 n-6 | 1.0 ± 0.3 | 0.5 ± 0.1 | 1.8 ± 0.9 | 0.3 ± 0.1 | 1.1 ± 0.4 | 0.7 ± 0.1 |
| 20:3 n-6 | 4.6 ± 1.3 | 4.1 ± 1.3 | 2.3 ± 0.8 | 4.2 ± 1.8 | 2.8 ± 0.7 | 3.8 ± 1.1 |
| 20:4 n-6 | 1.6 ± 0.4 | 1.7 ± 0.5 | 3.3 ± 0.7 | 1.5 ± 0.4 | 1.5 ± 0.3 | 1.5 ± 0.4 |
| 20:5 n-3 | 4.0 ± 0.7 | 2.8 ± 0.6 | 4.7 ± 0.6 | 4.0 ± 0.6 | 6.8 ± 0.6 | 2.9 ± 0.5 |
| 22:4 n-6 | 1.2 ± 0.5 | 0.6 ± 0.2 | 0.8 ± 0.4 | 1.3 ± 0.6 | 1.1 ± 0.4 | 0.4 ± 0.1 |
| 22:5 n-6 | 1.2 ± 0.2 | 1.1 ± 0.3 | 0.8 ± 0.2 | 1.9 ± 1.0 | 0.6 ± 0.3 | 1.5 ± 0.6 |
| 22:5 n-3 | 0.9 ± 0.1 | 0.7 ± 0.2 | 1.0 ± 0.2 | 0.5 ± 0.1 | 0.9 ± 0.2 | 0.7 ± 0.2 |
| 22:6 n-3 | 10.2 ±1.1 | 7.4 ± 1.6 | 8.7 ± 1.2 | 9.3 ± 1.3 | 10.3 ± 0.8 | 5.6 ± 0.7 |
| NMID 20:2 (Δ5, 11) | 4.5 ± 0.8 | 3.1 ± 0.8 | 3.7 ± 0.5 | 4.1 ± 0.7 | 7.5 ± 0.8 | 3.8 ± 0.8 |
| NMID 20:2 (Δ5, 13) | 1.7 ± 0.3 | 1.3 ± 0.3 | 2.2 ± 0.5 | 1.3 ± 0.2 | 1.4 ± 0.1 | 1.4 ± 0.2 |
| NMID 22:2 (Δ7, 13) | 0.7 ± 0.2 | 0.6 ± 0.1 | 0.9 ± 0.6 | 0.3 ± 0.1 | 0.7 ± 0.3 | 0.4 ± 0.1 |
| NMID 22:2 (Δ7, 15) | 0.1 ± 0.1 | 0.3 ± 0.2 | 0.3 ± 0.1 | 0.4 ± 0.2 | 0.0 ± 0.0 | 0.8 ± 0.7 |
| NMIT 22:3 (7, 13, 16) | 3.1 ± 1.3 | 3.6 ± 0.9 | 1.7 ± 0.7 | 3.5 ± 1.7 | 1.6 ± 0.5 | 4.2 ± 1.1 |
| DMA 16:0 | 0.2 ± 0.1 | 0.2 ± 0.0 | 0.2 ± 0.1 | 0.2 ± 0.1 | 0.2 ± 0.0 | 0.2 ± 0.1 |
| DMA 17:0 iso II | 0.1 ± 0.0 | 0.1 ± 0.0 | 0.1 ± 0.0 | 0.0 ± 0.0 | 0.1 ± 0.0 | 0.0 ± 0.0 |
| DMA 17:0 | 0.0 ± 0.0 | 0.1 ± 0.0 | 0.0 ± 0.0 | 0.0 ± 0.0 | 0.1 ± 0.0 | 0.1 ± 0.0 |
| DMA 18:0 iso I | 1.0 ± 0.4 | 1.0 ± 0.3 | 0.9 ± 0.3 | 0.7 ± 0.4 | 0.7 ± 0.3 | 0.1 ± 0.0 |
| DMA 18:0 | 1.8 ± 0.4 | 1.1 ± 0.3 | 1.7 ± 0.4 | 0.9 ± 0.3 | 2.3 ± 0.5 | 1.7 ± 0.5 |
| DMA 19:0 iso | 0.5 ± 0.1 | 0.4 ± 0.1 | 0.4 ± 0.1 | 0.1 ± 0.0 | 0.5 ± 0.1 | 0.2 ± 0.1 |
| DMA 20:1 iso II | 0.0 ± 0.0 | 0.0 ± 0.0 | 0.0 ± 0.0 | 0.0 ± 0.0 | 0.1 ± 0.1 | 0.3 ± 0.3 |
| DMA 20:1 | 8.0 ± 1.1 | 5.0 ± 1.2 | 7.2 ± 0.9 | 6.8 ± 1.0 | 9.3 ± 0.9 | 6.0 ± 0.8 |
| Branched FA | 2.5 ± 0.39 | 2.0 ± 0.3 | 2.1 ± 0.2 | 2.2 ± 0.4 | 1.7 ± 0.2 | 1.4 ± 0.2 |
| Fatty acids ˂ 0.5% | 3.4 ± 0.57 | 2.7 ± 0.4 | 4.0 ± 1.2 | 2.2 ± 0.4 | 3.6 ± 0.6 | 4.6 ± 1.4 |
| SFA | 45.6 ± 3.4 | 57.4 ± 6.3 | 49.5 ± 2.7 | 50.4 ± 2.0 | 41.5 ± 2.2 | 52.8 ± 3.5 |
| MUFA | 5.3 ± 0.4 | 4.8 ± 0.5 | 5.9 ± 0.4 | 6.6 ± 0.7 | 6.4 ± 0.3 | 6.3 ± 1.0 |
| PUFA | 36.6 ± 2.2 | 29.1 ± 4.2 | 33.6 ± 1.7 | 33.6 ± 1.9 | 38.1 ± 1.1 | 30.8 ± 3.0 |
| n-6 PUFA | 10.0 ± 1.5 | 8.4 ± 1.3 | 9.4 ± 1.4 | 9.5 ± 1.6 | 7.6 ± 0.9 | 8.3 ± 0.8 |
| n-3 PUFA | 15.8 ± 1.5 | 11.3 ± 2.3 | 15.1 ± 1.5 | 14.3 ± 1.7 | 19.0 ± 1.4 | 9.7 ± 1.2 |
| n-3 PUFA (%PUFA) | 44.3 ± 4.4 | 37.9 ± 4.6 | 45.0 ± 3.9 | 43.5 ± 5.0 | 49.7 ± 3.1 | 32.3 ± 4.1 |
| PUFA (without NMI) | 25.9 ± 1.5 | 19.7 ± 2.9 | 24.5 ± 1.1 | 23.8 ± 1.5 | 26.6 ± 1.4 | 18.0 ± 1.4 |
| NMI total | 10.8 ± 1.2 | 9.4 ± 1.3 | 9.1 ± 1.0 | 9.8 ± 1.1 | 11.5 ± 1.0 | 12.8 ± 2.0 |
| NMI (%PUFA) | 29.0 ± 2.0 | 32.7 ± 1.7 | 26.8 ± 2.2 | 29.1 ± 2.6 | 30.3 ± 2.8 | 40.4 ± 3.6 |
| DMA ˂ 0.5% | 0.0 ± 0.0 | 0.2 ± 0.2 | 0.1 ± 0.1 | 0.2 ± 0.1 | 0.0 ± 0.0 | 0.1 ± 0.1 |
| Branched DMA (iso) | 2.0 ± 0.6 | 2.0 ± 0.4 | 1.7 ± 0.4 | 1.2 ± 0.5 | 1.7 ± 0.5 | 1.7 ± 0.3 |
| DMA total | 12.4 ± 2.1 | 8.7 ± 1.7 | 10.9 ± 1.3 | 9.5 ± 0.6 | 14.0 ± 1.9 | 10.1 ± 1.5 |
| Unsaturation index | 160.9 ± 8.5 | 125.2 ± 19.2 | 148.4 ± 8.2 | 151.8 ± 8.0 | 170.1 ± 5.5 | 125.8 ± 9.9 |
| Peroxidation index | 145.0 ± 10.9 | 109.6 ± 18.6 | 138.1 ± 11.2 | 138.2 ± 12.2 | 157.0 ± 8.9 | 99.1 ± 6.9 |
| MRL (years) | 36 | 53 | 71 | 150 | 300 | 507 |
|  |  |  |  |  |  |  |
